# Human Hantavirus Infection, Brazilian Amazon

**DOI:** 10.3201/eid1207.060074

**Published:** 2006-07

**Authors:** Marcelo Cordeiro dos Santos, Marcus Vinícius Guimarães de Lacerda, Soledade Maria Benedetti, Bernardino Cláudio Albuquerque, Alfredo A. B. Vieira de Aguiar Filho, Mauro da Rosa Elkhoury, Elizabeth Salbé Travassos da Rosa, Pedro Fernando da Costa Vasconcelos, Daniele Barbosa de Almeida Medeiros, Maria Paula Gomes Mourão

**Affiliations:** * Tropical Medicine Foundation of Amazonas, Manaus, Amazonas, Brazil;; † Amazonas State University, Manaus, Amazonas, Brazil;; ‡ Nilton Lins University Center, Manaus, Amazonas, Brazil;; § University of Brasília, Brasília, Federal District, Brazil;; ¶ Health Surveillance Foundation, Manaus, Amazonas, Brazil;; # Ministry of Health, Brasília, Federal District, Brazil;; ** Evandro Chagas Institute, Belém, Pará, Brazil

**Keywords:** hantavirus, Brazil, case report, letter

**To the Editor:** Since hantavirus pulmonary syndrome (HPS) caused by Sin Nombre virus (SNV) was identified in the southwestern United States in 1993, cases have been diagnosed in many Latin American countries, and an increasing number of hantaviruses and their rodent reservoirs have been reported (*1*). The first evidence of hantavirus circulation in the western Brazilian Amazon region was documented in 1991 (*2*). Vasconcelos et al., by using antigens from the Old World hantavirus, found evidence of hantavirus antibodies in 45.2% of serum samples acquired from contacts of patients who died with undiagnosed hemorrhagic fever in Manaus.

The first human cases of symptomatic infection by hantaviruses were reported from Brazil in 1993, in Juquitiba (São Paulo State). HPS developed in 3 young brothers, who lived in a forested region along the Atlantic Coast, after they had cleared trees on their land, and 2 of them died. These patients were living in poor conditions, without appropriate storage spaces for human food or for animal feed, and their dwelling was constantly invaded by wild rodents who were looking for food (*3*). Since then, many other HPS cases have been reported, especially from the southern and southeastern regions of Brazil where agricultural activities are prominent; the mean case-fatality ratio is 48% (*3*). In the Brazilian Amazon, HPS has been frequently reported in Mato Grosso and sporadically in Maranhão and Pará states, which indicates an endemic circulation of hantaviruses (*4**,**5*)

We report here the first human cases of HPS in the state of Amazonas in the western part of the Brazilian Amazon. All 4 patients belonged to the same family cluster and came from a rural area near the town of Itacoatiara, on the edge of an important industrial waterway for soybean transport (the Itacoatiara soybean terminal). This family (patients 1, 2, and 3) had cleared a forested area on their farm and killed many rodents found in the bases of trees and near the house from May 25 to June 5, 2004. They also reported that wild rodents were inside their house.

All serologic tests were performed in the Arbovirology and Hemorrhagic Fever Department, at the Evandro Chagas Institute (Pará, Brazil), with antigens provided by the Centers for Disease Control and Prevention (Atlanta, GA, USA). An enzyme-linked immunosorbent assay (ELISA) was performed by using cellular fluid and Laguna Negra virus antigens for immunoglobulin M (IgM) detection (MAC-ELISA), and recombinant SNV antigens for IgG detection. Samples were considered positive with an optical density >0.2 in 1:100 (IgM) and 1:400 (IgG) dilutions (*6**,**7*). Viral isolation or polymerase chain reaction (PCR) for hantavirus were not attempted in human or rodent samples.

In the index patient, symptoms developed 15 days after she had killed 20 rodents with hot water during the tree-clearing process on the farm. She was a 25-year-old woman who sought treatment with an acute syndrome of high fever, dry cough, and dyspnea. She was admitted to the Itacoatiara general hospital; her condition was diagnosed as bacterial pneumonia and treated with intravenous penicillin. She died within 5 days because of respiratory failure; since no laboratory tests were conducted, she does not fulfill the case definition criteria for HPS. This was the only case in this series not confirmed with laboratory tests.

The second case was in the first patient's 31-year-old husband. Symptoms developed 2 weeks after the wife's death, starting with a 5-day febrile syndrome, which progressed to a dry cough and then respiratory distress, with a petechial rash, hemoconcentration, and thrombocytopenia (53,000 platelets/μL) over the next 2 days. He exhibited a diffuse, alveolar infiltrate on chest radiograph and a mild cardiomyopathy on echocardiogram. He was admitted to an intensive care unit and required mechanical ventilation for 10 days; he made a gradual recovery. Results of his laboratory tests ruled out malaria, dengue fever, and leptospirosis. Three consecutive blood culture samples were negative for bacterial growth. The IgG and IgM ELISA results for hantavirus were positive in both acute- and convalescent-phase serum samples.

The third case was in the second patient's brother, a 43-year-old man, who exhibited a self-limited, acute febrile syndrome 1 month after the index patient. He did not live on the same farm but visited there often and had actively participated in removing the trees on his brother's farm. He had no respiratory complaints, and results of his chest radiographs were normal, but the complete blood count showed hemoconcentration and mild thrombocytopenia (130,000 platelets/μL). He was hospitalized for 3 days and recovered completely. An IgM ELISA result was positive for hantavirus in 2 consecutive blood samples, and an IgG ELISA result was positive in convalescent-phase serum.

The fourth patient was a 67-year-old farmer, the uncle of the last 2 patients. He visited his nephew's farm regularly and was present during the deforestation process. He presented for medical assistance after a 15-day febrile syndrome, with a dry cough and mild dyspnea, 5 weeks after the index patient. He was hospitalized for 3 days and also had an uneventful recovery. The IgM ELISA result in this patient was also positive for hantavirus in 2 consecutive blood samples as was the IgG ELISA result for convalescent-phase serum.

Shortly after the report of the first 3 cases, the Brazilian Health Surveillance Secretary (Ministry of Health) performed an epidemiologic field study to seek the probable site of infection, collect sylvatic rodents, and conduct a serologic survey of human contacts. No areas for soybean cultivation or seed storage were found, but local farmers commonly store dry corn for feeding their domestic fowl. Eighty-two blood samples were collected from asymptomatic persons and were all negative for hantavirus IgG antibodies by ELISA. Eleven sylvatic rodents were captured in 3 days of trapping (270 traps/night) on the farm, on neighboring farms, and in the nearby forest. Two species were identified, *Proechimys* sp. (2 animals) and *Oligoryzomys microtis* (9 animals). This finding is very similar to reports of rodents in other regions of Brazil (*8*). Four *Oligoryzomys microtis* had positive IgG results for hantavirus (*9*).

Identification of human and rodent hantavirus infection in the Amazonas State adds this emergent disease to our differential diagnoses of febrile tropical diseases and to our syndromic surveillance approach for febrile respiratory diseases. Further research is needed to identify the viral genotype that circulates in this area and to determine the real prevalence of human infection and the epidemiologic scenario of HPS in the western Brazilian Amazon region.
